# Genomic imprinting effects on complex traits in domesticated animal species

**DOI:** 10.3389/fgene.2015.00156

**Published:** 2015-04-24

**Authors:** Alan M. O’Doherty, David E. MacHugh, Charles Spillane, David A. Magee

**Affiliations:** ^1^UCD Conway Institute of Biomolecular and Biomedical Research, University College Dublin, BelfieldIreland; ^2^Animal Genomics Laboratory, UCD School of Agriculture and Food Science, University College Dublin, BelfieldIreland; ^3^Genetics and Biotechnology Laboratory, Plant and AgriBiosciences Research Centre, School of Natural Sciences, National University of Ireland Galway, GalwayIreland; ^4^Department of Animal Science, University of Connecticut, Storrs, CTUSA

**Keywords:** complex traits, epigenetics, epigenome, genomic imprinting, livestock

## Abstract

Monoallelically expressed genes that exert their phenotypic effect in a parent-of-origin specific manner are considered to be subject to genomic imprinting, the most well understood form of epigenetic regulation of gene expression in mammals. The observed differences in allele specific gene expression for imprinted genes are not attributable to differences in DNA sequence information, but to specific chemical modifications of DNA and chromatin proteins. Since the discovery of genomic imprinting some three decades ago, over 100 imprinted mammalian genes have been identified and considerable advances have been made in uncovering the molecular mechanisms regulating imprinted gene expression. While most genomic imprinting studies have focused on mouse models and human biomedical disorders, recent work has highlighted the contributions of imprinted genes to complex trait variation in domestic livestock species. Consequently, greater understanding of genomic imprinting and its effect on agriculturally important traits is predicted to have major implications for the future of animal breeding and husbandry. In this review, we discuss genomic imprinting in mammals with particular emphasis on domestic livestock species and consider how this information can be used in animal breeding research and genetic improvement programs.

## Introduction

Mammals are diploid organisms characterized by the presence of complete sets of paternally- and maternally inherited chromosomes in each somatic cell. Normal mammalian development requires that the paternal and maternal copy of each gene (i.e., parental alleles) is expressed correctly, with each copy having the potential to be expressed equally (e.g., to the same level) in each cell. However, a subset of mammalian autosomal genes has been identified where expression is restricted to one of the two parentally inherited chromosomes in a parent-of-origin specific manner; such genes are said to be imprinted. Imprinted genes on autosomal chromosomes can affect both male and female offspring, and such imprinting effects do not arise as a consequence of sex chromosome inheritance. Rather, ‘classically defined’ autosomal imprinting is a consequence of the parental origin of each allele such that, in general, paternally expressed/maternally imprinted genes are transcriptionally silenced on the maternally inherited chromosome only, while maternally expressed/paternally imprinted genes are silenced solely on the paternally inherited chromosome (Barlow and Bartolomei, 2014). Not all imprinted genes adhere to this classic definition; for some genes transcriptional repression of the ‘imprinted’ parental allele is partial (sometimes termed ‘preferential’ or ‘allele-specific’ gene expression) wherein one allele displays higher levels of expression relative to the other allele in a parent-of-origin manner, while other genes display tissue-and/or temporal-specific imprinting or imprinting patterns that differ between individuals of the same species (Giannoukakis et al., 1996; Prickett and Oakey, 2012).

Importantly, mammalian genes displaying genomic imprinting are distinguishable from genes that display apparent parental-specific expression due to unequal or unique genetic contributions from male and female parents such as the expression of Y-linked genes in XY males, the expression of maternally derived mitochondrial genes, and the expression of X-linked genes that evade the process of X-chromosome inactivation in XX females. X-chromosome inactivation, in particular, has been extensively studied in mammals since it was first described by Lyon (1961). During early female embryonic development, one of the two X-chromosomes is randomly inactivated to equalize the X-linked gene dosage difference between XX females and XY males. This process, called ‘random X-inactivation’, involves the decoration of one X-chromosome with a non-protein coding RNA (termed *XIST*), which initiates the chromosome-wide gene silencing of the X-chromosome from which the *XIST* transcript is derived. Interestingly, preferential inactivation of the paternally derived X-chromosome involving *XIST* transcripts has been reported in the placental tissue of XX female mammals, a process known as ‘imprinted X inactivation’ (Chow and Heard, 2010; Lee and Bartolomei, 2013).

Genomic imprinting was first described ~30 years ago through pronuclear transplantation experiments (Barton et al., 1984; Surani et al., 1984; Cattanach and Kirk, 1985). This work demonstrated that normal murine embryo development requires genetic contributions from both the maternally and paternally inherited haploid genomes. Diploid mouse embryos reconstructed from two maternal or paternal pronuclei with no genetic contributions from paternal or maternal sources (i.e., gynogenetic and androgenetic embryos, respectively) failed to survive. It was hypothesized that a subset of murine genes, expressed solely from the maternal- or paternal-derived haploid genomes, was necessary for normal embryonic growth and development and that these genes carry specific epigenetic marks or ‘imprints’ that control this parent-of-origin, monoallelic expression (Barton et al., 1984; Surani et al., 1984; Cattanach and Kirk, 1985). Gynogenetic and androgenetic embryos have also been generated for cattle, sheep and pigs with results revealing arrested fetal development and lethality, due to aberrant genomic imprinting patterns (Fukui et al., 1992; Lagutina et al., 2004; Zacchini et al., 2011; Sembon et al., 2012).

To date, 132 murine and 79 human imprinted genes (including protein-coding and regulatory non-coding RNA genes) have been documented; however, only 25, 21, and 14 experimentally validated imprinted genes/loci have been reported for cattle, pigs and sheep, respectively (Morison et al., 2001; Jirtle, 2013; Wei et al., 2014). Initial evolutionary studies suggested that imprinted genes were largely conserved across mammalian species (Morison et al., 2005); however, more recent studies have shown that conservation of imprinted genes between primates and rodents is more limited than initially thought (Monk et al., 2006; Khatib et al., 2007). For example, of the 79 imprinted human genes reported in the *MetaImprint* database (Wei et al., 2014) only 40 of these (51%) are among the 132 imprinted genes reported for mice. Despite this limited conservation, imprinted genes have been shown to share a number of defining features among mammals. For instance, functional analyses have shown that many imprinted genes encode products that regulate a wide range of biological processes—most notably, embryonic and neonatal growth and development, metabolism and behavior—in all mammalian species studied to-date (Plasschaert and Bartolomei, 2014; Tian, 2014). Furthermore, while some imprinted genes map as singletons or as gene pairs, many imprinted genes are organized into clusters (~1 Mb) in which both maternally- and paternally imprinted genes (including protein- and RNA-coding genes) reside and whose expression is regulated by a discrete region [termed ‘the imprinting control region’ (ICR)] located within the clusters (Barlow and Bartolomei, 2014).

The important regulatory roles of ICRs has been highlighted in human biomedical studies, whereby epigenetic or genetic alterations (e.g., DNA sequence changes, deletion of an ICR, loss or gain of an imprint) at these sites result in dysregulated expression of reciprocally imprinted genes leading to developmental disorders (Edwards and Ferguson-Smith, 2007). In cattle, deletion of a 110 kb region proximal to the ICR regulating the expression of the paternally expressed/maternally imprinted *PEG3* domain was recently shown to result in the loss of paternal *MIMT1* expression in the brain and cotyledon of all carrier fetuses. This mutation is thought to be responsible for late fetal mortality and stillbirth in 85% of the offspring inheriting the causative mutation from the founding sire; it has been postulated that the remaining 15% of progeny inheriting the mutation survive due to incomplete silencing of the maternally inherited *MIMT1* allele (Flisikowski et al., 2010, 2012).

The co-localization of imprinted genes has resulted from the processes by which these loci are hypothesized to have evolved. The most credible explanation with significant supporting evidence is the ‘*conflict theory*’ of genomic imprinting, which states paternally expressed imprinted genes (e.g., *IGF2*) have evolved to actively promote fetal growth and development, thereby maximizing maternal resources to offspring bearing a particular paternal genome during gestation. In contrast, maternally expressed imprinted genes (e.g., *IGF2R*) have evolved to suppress fetal growth, thereby causing a more uniform distribution of maternal resources to all offspring carrying a particular maternal genome, despite possessing different paternal genomes (Moore and Haig, 1991; Ashbrook and Hager, 2013).

## Genomic Imprinting is a Form of Epigenetic Regulation

Genomic imprinting is an epigenetic mechanism of gene expression regulation, whereby alterations in gene expression do not involve any changes to underlying DNA sequences. Epigenetic regulation is largely characterized by the regional addition or removal of a chemical imprint to either genomic DNA (e.g., DNA methylation) and/or chromatin-associated proteins (e.g., histone acetylation, methylation, ubiquintination). Such epigenetic “imprints” can serve to mediate the local expression of genes, either through transcriptional activation, transcriptional attenuation or complete transcriptional silencing. In mammals, parent-of-origin-specific expression due to genomic imprinting is reliant on the existence of epigenetic differences between the two parental alleles resulting in their differential expression in the same nucleus (Hanna and Kelsey, 2014; Weaver and Bartolomei, 2014).

Genomic imprinting involves the establishment of differential imprints on chromosomes inherited either via the male or the female germ lines during meiosis according to their parent-of-origin. Importantly, such differential imprints are reversible, whereby an imprint established on a chromosome inherited via the female germline will not be established when the same chromosome is inherited via the male germline (or *vice versa*) in the subsequent generation. These parent-of-origin imprints can be then inherited by all daughter cells through mitosis following fertilization, potentially resulting in imprinted gene expression patterns throughout the lifespan of the animal (Abramowitz and Bartolomei, 2012).

For epigenetic regulation of the imprinted status of genes, the epigenetic imprint must exhibit four major mechanistic attributes: firstly, the imprint must be able to regulate gene product levels; secondly, the imprint must be stably inherited in somatic cells such that the ‘memory’ of parental origin is faithfully transmitted to daughter cells during mitosis; thirdly, the imprint is established independently on either the paternal or maternal genomes when they are not present in the same nucleus (e.g., during meiosis); and fourthly, the imprint must be erased and reset in the germ line such that appropriate parent-of-origin identity is established in the gametes for the subsequent generation (Bartolomei, 2009).

Although many diverse biomolecular mechanisms are now classified as epigenetic (e.g., histone tail modifications and expression of small and long non-coding RNAs), the most extensively studied epigenetic marks associated with genomic imprinting is DNA methylation (Kelsey and Feil, 2013; Plasschaert and Bartolomei, 2014). In mammals, DNA methylation involves the addition of a methyl group (-CH_3_) by DNA methyltransferase enzymes to the 5′ carbon of cytosine residues that exist primarily in CpG dinucleotides [i.e., cytosine-phosphate-guanine residues that lie adjacent to each other on the same DNA strand] (Bird, 2007). Cytosine methylation at CpG dinucleotides has been shown to be associated with imprinted gene regulation, particularly at genomic regions where CpGs located in promoter-associated and non-promoter-associated ICRs display differential methylation patterns on both the maternally and paternally inherited chromosomes [i.e., differentially methylated regions (DMRs; Henckel et al., 2009; Ito et al., 2013)].

**FIGURE 1:**
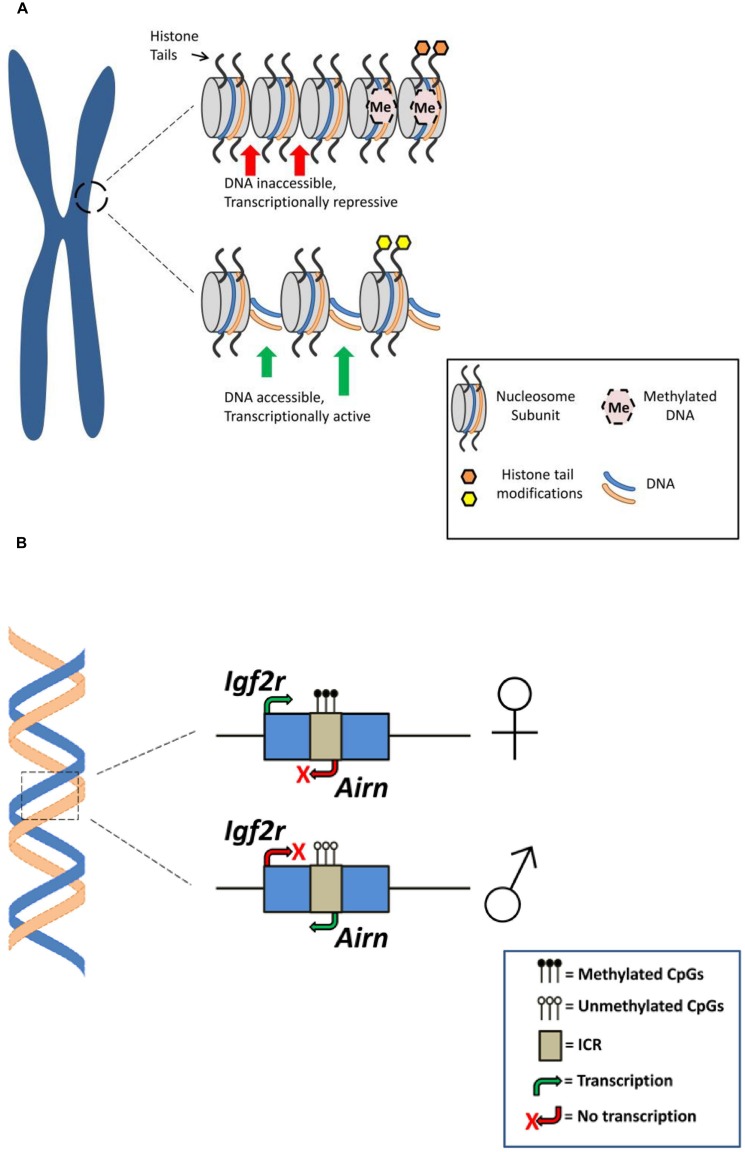
**Epigenetic mechanisms associated with genomic imprinting. (A)** Histone modifications and DNA methylation for different chromatin configurations. Top: Repressive chromatin state associated with histone modification (e.g., histone methylation; orange shading) and dense DNA methylation resulting in gene silencing or attenuated gene expression. Bottom: Active/permissive chromatin state associated with histone modification (e.g., histone acetylation; yellow shading) and reduced DNA methylation rendering DNA accessible for transcription resulting in gene expression (for a comprehensive overview of histone modifications see Bannister and Kouzarides, 2011). **(B)** Genomic arrangement at an imprinted gene. A simplified schematic of the murine *Igf2r* locus demonstrating parent-of-origin specific DNA methylation is presented. The imprinting control region (ICR) on the maternal *Igf2r* allele is methylated, preventing expression of an antisense ncRNA (*Airn*) and resulting in expression of the maternal *Igf2r* allele. Alternatively, expression of *Airn* from the unmethylated paternal allele attenuates paternal *Igf2r* expression (for a more comprehensive overview of DNA methylation at the *Igf2r* locus and genomic imprinting see Autuoro et al., 2014).

DNA methylation is widely considered as a repressive gene expression mechanism that regulates imprinted gene expression by promoting chromatin condensation, rendering the DNA less accessible to the cell’s transcriptional machinery (**Figure 1**). Thus, silenced or repressed gene expression is generally observed from the hypermethylated DMR (Hanna and Kelsey, 2014). For example, a recent survey of the allelic methylation profile of human genes in placental tissue revealed that for a panel of known paternally expressed imprinted genes the promoters are methylated on the maternal allele, with corresponding allele-specific expression from the paternal allele (Court et al., 2014). In addition to its classical role in repression of gene expression there is a growing body of evidence demonstrating that DNA methylation, particularly within intragenic regions, may be involved with promoting transcription (Neri et al., 2013; Irwin et al., 2014).

Post-translational modifications of histone proteins are also recognized as an important epigenetic regulatory mechanism associated with mammalian imprinted genes (**Figure 1**). In eukaryotic cell nuclei, DNA is tightly packed into chromatin such that the DNA double-helix is wrapped around the histone octameric core to form the basic chromatin unit, the nucleosome. The N-terminal regions of histone proteins that protrude from the nucleosome can undergo various post-translational modifications (e.g., methylation, acetylation, ubiquitination ,and phosphorylation) that can regulate gene expression (Weaver and Bartolomei, 2014). For example, acetylation and methylation of histone lysine residues—typically associated with transcriptionally active and repressed chromatin, respectively—has been associated with several murine imprinted genes including the linked and reciprocally-imprinted *H19* and *Igf2* genes and the genes located in the *Kcnq1* imprinted cluster on chromosome 7 (Pedone et al., 1999; Wagschal et al., 2008; Ciccone et al., 2009).

RNA-mediated gene expression regulation is an additional epigenetic mechanism that is pertinent to understanding the regulation of imprinted gene expression. Epigenetic regulation by long non-coding RNAs (ncRNAs) is well established for X-chromosome inactivation in female mammals (Briggs and Reijo Pera, 2014). Long ncRNAs have also been implicated in the regulation of imprinted loci in mammals, in conjunction with other molecular mechanisms such as insulators, DNA methylation, and histone modifications (Barlow and Bartolomei, 2014). In contrast to *trans*-acting RNA interference (RNAi)-mediated repression of gene expression, macro ncRNAs, (which are often 100s of kb in length) can elicit *cis*-regulatory effects on gene expression, and thereby can generate allele-specific imprinting effects on gene expression (Koerner et al., 2009).

In general, studies of imprinted gene regulation and imprinted gene clusters are revealing a complex interplay between DNA methylation, histone modifications, higher-order chromatin structure, RNA-mediated epigenetic effects and transcription, which are all involved in the establishment of the primary genomic “imprint” (Koerner et al., 2009; Adalsteinsson and Ferguson-Smith, 2014).

## Epigenetic Dynamics During Mammalian Gametogenesis and Early Development

DNA methylation provides an example of an epigenetic mark that is highly dynamic and that can undergo spatio-temporal changes across cells, tissues and generations (Schneider et al., 2010). Much of what is known regarding DNA methylation dynamics during development comes from studies in mice. Dramatic genome-wide changes in DNA methylation occur during gametogenesis and the early stages of embryo development (Reik and Walter, 2001; Messerschmidt et al., 2014). Primordial germ cells (PGCs) are almost completely ‘erased’ of DNA methylation marks upon entry into the genital ridge (Hajkova et al., 2002; Seisenberger et al., 2012), with some single-copy loci and transposable elements, such as intracisternal A-particles (IAP) and certain endogenous retroviral-derived sequences, retaining moderate levels of methylation (Lane et al., 2003; Guibert et al., 2012). Following this, gamete-specific methylated regions are established during spermatogenesis and oogenesis and these patterns substantially differ depending on which germline they occur. Such methylation differences are most noticeable at imprinted loci whereby specific genomic regions become asymmetrically methylated in sperm and oocytes. In the male germline, imprinted genes can acquire their gamete-specific DNA methylation marks in fetal prospermatagonia prior to birth (Davis et al., 2000; Li et al., 2004). This period of *de novo* methylation has also been shown to coincide with global changes in histone tail modifications, which are not observed in female germ cells during this period of development (Yoshioka et al., 2009; Singh et al., 2013). Maternal-specific DNA methylation at imprinted genes is acquired in the postnatal growing oocyte (Hiura et al., 2006; O’Doherty et al., 2012).

Following fertilization there is a global cascade of DNA demethylation during the early stages of embryogenesis, whereby the paternal genome is rapidly demethylated in the zygote and the maternal genome is passively demethylated in a replication-dependent manner (Dean et al., 2001; Yang et al., 2007; Iqbal et al., 2011). More recently, it has been hypothesized that both the maternal and paternal genomes undergo global active demethylation and replication-mediated passive demethylation (Gkountela and Clark, 2014; Guo et al., 2014). Irrespective of the mechanisms controlling these genome-wide reprogramming events in the pre-implantation embryo, DNA methylation at imprinted genes is generally considered as being stable until they undergo reprogramming in PGCs (Olek and Walter, 1997; Imamura et al., 2005; Smallwood et al., 2011). However, a study analyzing imprinted DMRs in mouse blastocysts revealed dynamic changes in allelic methylation, suggesting that DMRs are not fully protected from the major reprogramming events in the early embryo (Tomizawa et al., 2011).

## Epigenetic Programming and Imprinted Disorders in Domestic Livestock Species

In domesticated species, the importance of establishing appropriate epigenetic marks at imprinted loci has been highlighted largely through assisted reproductive technologies (ART) including somatic cell nuclear transfer (SCNT) cloning studies. ART involves the isolation, handling, manipulation and culture of gametes, and early embryos, usually after hormonal stimulation. As discussed above, major epigenetic reprogramming events occur during gametogenesis and early embryonic development and it has been proposed that ART exposes the epigenome to external factors that may interfere with the correct establishment and maintenance of genome imprints. For example, superovulation, embryo culturing and cryopreservation can affect methylation profiles and gene expression at imprinted loci (Humpherys et al., 2001; DeBaun et al., 2003; Gicquel et al., 2003; Kang et al., 2003; Chang et al., 2005; Ludwig et al., 2005; Sato et al., 2007). Epigenetic perturbations, associated with ART and SCNT, may contribute to developmental issues such as increased abortion rate, perinatal death, enlarged placentomes, enlarged umbilical cords, high-birth weight and large offspring syndrome (LOS; Campbell et al., 1996; Cibelli et al., 1998; Kang et al., 2003; Alexopoulos et al., 2008; Smith et al., 2012).

Another example of an epigenetic-associated developmental disorder is LOS. LOS is an overgrowth disorder in domesticated ruminants bearing phenotypic similarities to Beckwith–Wiedemann syndrome (BWS, an overgrowth disorder in humans), and is characterized by excessive birth weight, enlarged tongue, umbilical hernia, enlarged internal organs and hypoglycemia (Young et al., 1998; Weksberg et al., 2010). Both BWS and LOS can occur naturally; however, there is evidence that these disorders have an increased incidence in individuals generated from ART (Chang et al., 2005).

Previous work has shown that epigenetic changes (also referred to as ‘epimutations’) at two ICRs, that independently regulate the expression of two clusters of reciprocally imprinted genes on human chromosome 11p15, are associated with BWS (Choufani et al., 2010). One imprinting cluster contains the maternally expressed/paternally imprinted ncRNA *H19* gene and the paternally expressed/maternally imprinted *IGF2* gene, which encodes a fetal mitogen. Studies have shown that both genes are under the control of a single ICR that is unmethylated on the maternal allele and methylated on the paternal allele. In mice, binding of the CCCTC-binding factor (zinc finger protein), CTCF, to the non-methylated ICR inhibits maternal expression of *Igf2* by preventing interaction of its promoter with downstream enhancers; however, the *H19* promoter has access to the downstream promoters resulting in its maternal expression (Hark et al., 2000; Demars et al., 2010; Poole et al., 2012). The second cluster contains a paternally expressed ncRNA gene, *Kcnq1ot1*, and several maternally expressed protein-coding genes associated with regulating growth and development, such as *Cdkn1c*, *Kcnq1*, and *Phlda2*. Expression of the genes in this cluster is controlled by a single ICR known as KvDMR1, which is hypomethylated on the paternal copy (Fitzpatrick et al., 2002; Choufani et al., 2010). Paternal expression of *Kcnq1ot1* recruits the binding of Polycomb group proteins and initiates histone-tail methylation, which induces a transcriptionally repressive chromatin structure leading to silencing of the protein-coding genes from this locus on the paternal chromosome. Conversely, methylation of the KvDMR1 on the maternal allele prevents *Kcnq1ot1* transcription, thus, enabling the protein-coding genes to be expressed from the maternal allele (Fitzpatrick et al., 2002; Pandey et al., 2008; Terranova et al., 2008; Redrup et al., 2009; Choufani et al., 2010). In humans, gain-of-methylation epimutations at the maternal *IGF2*/*H19* ICR, resulting in increased expression of *IGF2*, can account for 2–7% of all BWS cases, while 50% of cases are due to loss-of-methylation epimutations at the maternal ICR (known as KvDMR1), which is concomitant with biallelic expression of *KCNQ1OT1* and downregulation of *CDKN1C*, a negative regulator of cell proliferation (Weksberg et al., 2001, 2010).

Similarly, studies in ruminants have revealed associations between aberrant methylation at the *H19-IGF2* and the *KCNQ1OT1*-*CDKN1C* loci and ART-generated fetuses, especially in offspring displaying LOS or which had died shortly after birth (Young et al., 1998; Hiendleder et al., 2004; Farin et al., 2006). For example, investigation of the DNA methylation status within the bovine *IGF2*-*H19* ICR revealed hypomethylation in several cloned animals relative to control animals, which correlated with biallelic expression of *H19* in the liver and placenta of these animals (Curchoe et al., 2009). Biallelic expression of bovine *IGF2* has also been observed in the brain and spleen tissue of ART-generated animals displaying LOS (Chen et al., 2013). Loss of maternal KvDMR1 methylation has also been associated with biallelic expression of *KCNQ1OT1* and reduced expression of *CDKN1C* in LOS bovine calves and fetuses, suggesting similarities in the epigenetic mechanisms that underlie both BWS and LOS (Hori et al., 2010; Chen et al., 2013). Furthermore, Young et al. (2001) also demonstrated that sheep fetuses displaying LOS has reduced maternal *IGF2R* mRNA and protein levels relative to control fetuses, which was correlated with a loss of methylation at the *IGF2R* ICR on the maternally active allele. In mice, the *Igf2r* ICR contains an antisense ncRNA, *Airn*, which when expressed from the unmethylated paternal allele attenuates paternal *Igf2r* expression (Latos et al., 2009, 2012). Thus, for LOS sheep it is conceivable that loss of *IGF2R* ICR methylation on the maternal chromosome results in increased transcriptional activity from the *AIRN* promoter leading to a corresponding reduction in *IGF2R* mRNA and protein expression (Bartolomei, 2009). Also, the bovine *IGF2R* gene has also been shown to have a maternally methylated DMR located in the second intron (O’Doherty et al., 2012), which displays reduced methylation in both ART- and SCNT-derived samples relative to *in vivo* samples (Smith et al., 2012); it is possible that this locus, and expression of the ncRNA *AIRN*, may be disrupted in bovine LOS.

## The Complex Interplay between Genetic and Epigenetic Mechanisms in Regulating Gene Expression: the Callipyge Phenotype in Sheep

In the context of genomic imprinting, individual epigenetic regulatory mechanisms do not function independently. Rather, multiple mechanisms tend to work in concert to define the functional states of chromatin that are associated with the regulation of imprinted gene expression (Jones et al., 1998). For example, ICRs displaying differentially methylated DNA regions are often also associated with transcriptionally repressive histone modifications such as methylated lysine residues resulting in chromatin condensation and silenced or repressed gene expression (Yang et al., 2003; Delaval et al., 2007; Meissner et al., 2008). Indeed, data from studies in mouse have led to the proposal that DNA methylation recruits repressive histone modifications at ICRs, thereby suggesting a positive feedback loop for the establishment and maintenance of parental imprints during development (Henckel et al., 2009).

**FIGURE 2:**
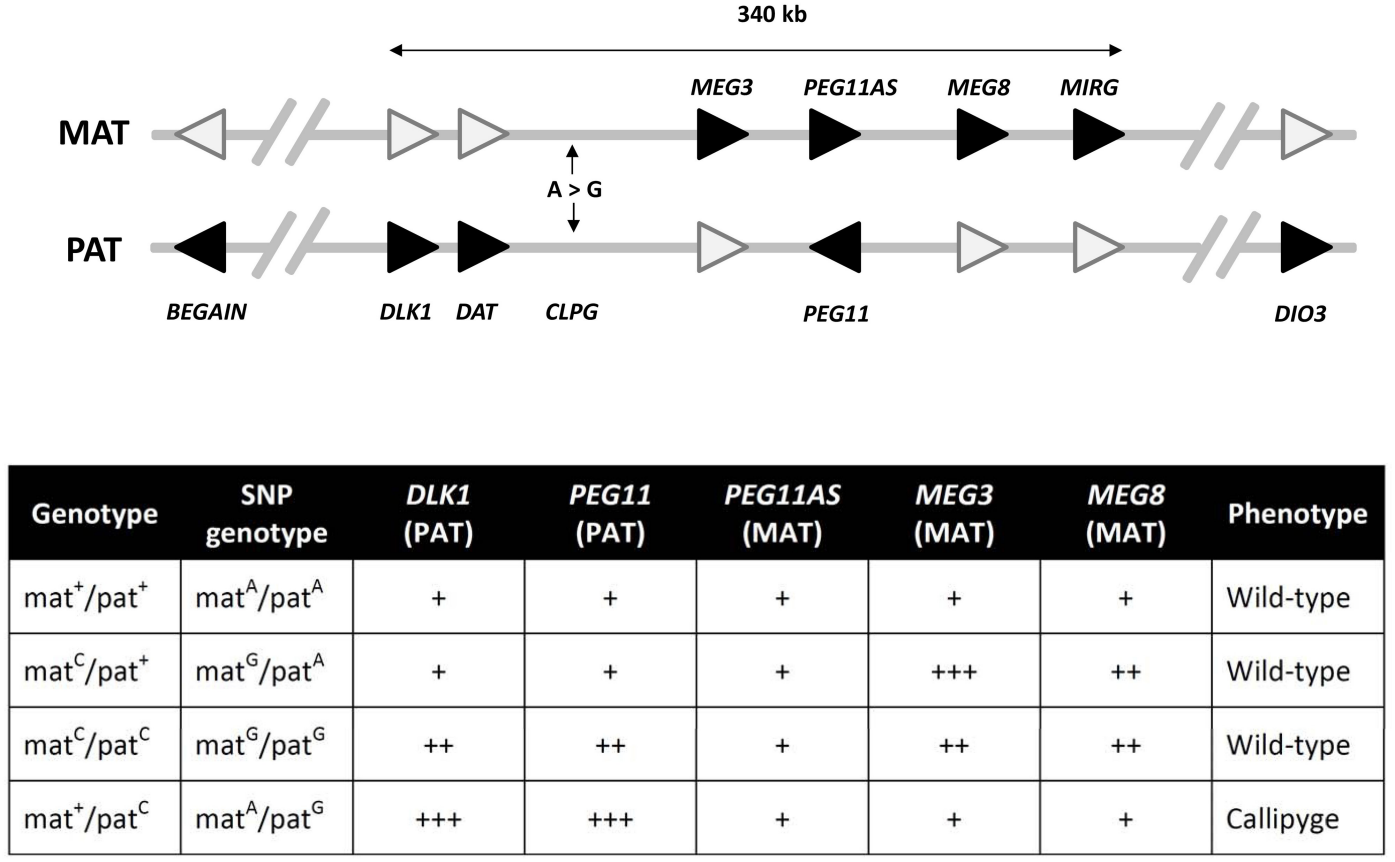
**The *DLK1*-*DIO3* imprinting domain on ovine chromosome 18**. This domain contains the genes whose expression is perturbed upon inheritance of the callipyge mutation (*CLPG*; an A-to-G SNP). The genes shaded in black represent the expressed imprinted alleles within this domain while white shading indicates the silenced/attenuated imprinted allele on either the maternal (MAT) or paternal (PAT) chromosomes. The arrowhead denotes the direction of transcription of each gene. Genes are not drawn to scale and introns are not shown. The core imprinted genes that have been shown to play a role in the callipyge phenotype occur within a 340 kb region. The expression of the core genes for each of the four possible callipyge genotypes at the *CLPG* SNP and the observed is summarized in the accompanying table. The relative RNA transcript abundance for the paternally (*DLK1*, *PEG11*) and maternally (*PEG11AS*, *MEG3*, *MEG8*, and *MIRG*) expressed genes are shown (not to scale) for each callipyge genotype. Callipyge animals (mat^+^/pat^C^) exhibit overexpression of *DLK1* and *PEG11* and an absence of *MEG3* and *MEG8* overexpression suggesting that *DLK1* and/or *PEG11* encodes the primary effector of the callipyge phenotype. Overexpression of the maternal non-coding RNA genes and the absence of muscle hypertophy in mat^C^/pat^C^ animals suggest that these transcripts exert their effect via post-transcriptional suppression of the effector. The microRNAs encoded by *MIRG* have been postulated to also play a role in post-transcriptional suppression of the paternally expressed effector (Georges et al., 2003; Bidwell et al., 2004, 2014; Murphy et al., 2006).

The complex interplay between different epigenetic and genetic mechanisms in regulating mammalian imprinted gene expression is aptly illustrated by the callipyge phenotype in sheep, which is responsible for a ~30% increase in skeletal muscle (most notably at the hindquarters), a corresponding ~8% reduction in fat content and improved feed efficiency (Cockett et al., 1996). This phenotype is observed only in heterozygous individuals that carry the causative mutation on the paternal chromosome (i.e., mat^+^/pat*^C^*, where ‘mat’ and ‘pat’ denote maternal and paternal chromosomes, respectively and superscript ‘+’ and ‘*C*’ represent wild-type and callipyge alleles, respectively)—a mode of non-Mendelian inheritance termed ‘polar overdominance’ (Cockett et al., 1996). The callipyge phenotype is caused by an A-to-G single nucleotide polymorphism (SNP; i.e., the callipyge mutation) located between the paternally expressed/maternally imprinted *DLK1* protein-coding gene and the maternally expressed/paternally imprinted *MEG3* long non-coding RNA (ncRNA) gene within the imprinted *DLK1-DIO3* gene cluster on ovine chromosome 18 (Freking et al., 2002; Smit et al., 2003). This cluster also contains additional paternally expressed/maternally imprinted protein-coding genes such as *PEG11*, and several maternally expressed/paternally imprinted long ncRNA and microRNA (miRNA) genes (including *MEG3*, *PEG11AS*, *MEG8*, and *MIRG* (also referred to as *MEG9*; Freking et al., 2002; Smit et al., 2003; Hagan et al., 2009; **Figure 2**).

Callipyge individuals (i.e., mat^+^/pat*^C^*) display overexpression of the paternally expressed *DLK1* and *PEG11* protein-coding transcripts in skeletal muscle tissue relative to non-callipyge animals (i.e., mat^+^/pat^+^; mat*^C^*/pat^+^; mat*^C^*/pat*^C^*). In contrast, individuals that inherit the callipyge mutation on the maternal chromosome (i.e., mat*^C^*/pat*^C^* or mat*^C^*/pat^+^) display upregulation of maternal long ncRNAs and miRNAs *in cis* relative to wild-type (i.e., mat^+^/pat^+^) and callipyge animals (Murphy et al., 2006). The callipyge mutation also causes a muscle tissue-specific reduction of methylation at CpG sites distributed across the *DLK1-DIO3* imprinted cluster resulting in increased transcriptional activity from the parental chromosome carrying the mutation (Takeda et al., 2006). Downregulated expression of the histone deacetylase 9 (*HDAC9*) gene—the encoded product of which removes acetyl groups from histone proteins resulting in increased chromatin condensation and repressed transcription—has also been observed in callipyge animals relative to non-callipyge animals suggesting a role for histone modification in regulation of expression at the *DLK1-DIO3* imprinted cluster (Vuocolo et al., 2007). Consequently, the callipyge mutation modifies the chromatin structure of the chromosome on which it is carried, such that the DNA surrounding the callipyge mutation is more permissive for transcription (Murphy et al., 2006).

A recently refined model of the polar overdominance observed for the callipyge phenotype suggests that *DLK1* and/or *PEG11* are likely to be the primary effectors of the callipyge phenotype—it is possible that the encoded products of *DLK1* and *PEG11* may act synergistically (Georges et al., 2003; Bidwell et al., 2004, 2014). Inheritance of the callipyge mutation on the paternal chromosome results in chromatin relaxation in the vicinity of the *DLK1-DIO3* imprinted cluster leading to increased *DLK1* and/or *PEG11* expression, which induces the hypertrophy response. Conversely, bi-parental inheritance or maternal inheritance of the callipyge mutation results in the upregulation of the *DLK1-DIO3* maternally expressed/paternally imprinted ncRNA and miRNA genes relative to callipyge and wild-type animals. This leads to repression of the phenotypic effects of *DLK1* and/or *PEG11* and many other genetic loci that regulate the hypertrophy response, thus, giving rise to the normal phenotype (Bidwell et al., 2014). Furthermore, it has been proposed that the repressive activity of the maternal long ncRNAs and miRNAs is achieved by inhibiting the expression of genes and proteins (at the transcriptional and/or post-transcriptional level through RNAi) involved in hypertrophy. In support of this, it has been shown that *PEG11AS* encodes six miRNAs that promote RNA-induced silencing complex (RISC)-mediated cleavage of *PEG11* transcripts (Davis et al., 2005); however, miRNA transcripts generated from the *DLK1-DIO3* imprinted cluster have been reported to not mediate the expression of *DLK1* (Cheng et al., 2014). It has also been suggested that maternally-derived *DLK1-DIO3* miRNAs may act to stabilize in *trans* the expression of several ncRNA transcripts that regulate hypertrophy (Tellam et al., 2012; Bidwell et al., 2014; **Figure 2**).

Molecular analysis of the callipyge phenotype highlights the role played by genetic (i.e., the A-to-G SNP that defines the callipyge mutation) and epigenetic mechanisms (i.e., DNA methylation, histone modifications, RNAi mechanisms and chromatin remodeling that regulate the expression of the genes within the *DLK1*-*DIO3* imprinting domain) in regulating complex phenotypes. Indeed, the callipyge phenotype demonstrates that the mammalian ‘hard-wired’ genome is not the single repository of regulatory information that has phenotypic effects, but that the ‘soft-wired’ epigenome—the collective term for epigenetic mechanisms that regulate gene expression—also has an important role in determining phenotype (Hanna and Kelsey, 2014).

## Imprinted Genes are Associated with Complex Phenotypic Traits in Mammals

Although analysis of mammalian genomes have shown that <1% of the total number of known mammalian protein-coding genes (~100 genes based on current versions of the human, mouse and bovine genomes in the Ensembl database) are subject to imprinting, several of these have been shown to have major effects on complex mammalian phenotypes. In mice, for example, studies have demonstrated the contribution of imprinted genes to variation in adiposity and body weight, muscle traits, metabolism, and disease susceptibility and resistance to infectious disease (Leighton et al., 1995; York et al., 1997; Clapcott et al., 2000; Lawson et al., 2011). Genetic studies of human phenotypes have also implicated imprinted gene effects in many biomedical conditions including BWS, Prader-Willi and Angelman syndromes, Alzheimer’s disease, cancer and type II diabetes (Bassett et al., 2002; Kong et al., 2009; Bird, 2014; Chaudhry et al., 2014; Eggermann et al., 2014). Similarly, while investigations of the callipyge phenotype have demonstrated a role for imprinting in sheep muscle traits, studies in pigs have identified a single SNP (G-to-A mutation) in the paternally expressed/maternally imprinted porcine *IGF2* gene that is responsible for ~30% of the variance for lean meat, 15–30% of the variance for muscle mass and 10–20% of the variance for backfat content (Jeon et al., 1999). This SNP was shown to be located in an evolutionarily conserved CpG island within *IGF2* intron 3 that abrogates binding of the zinc finger, BED-type containing 6 (ZBED6) transcriptional repressor. Animals inheriting a sire-derived ‘A’ nucleotide display a three-fold increase in *IGF2* expression in post-natal muscle relative to those animals inheriting a sire-derived ‘G’ nucleotide, which results in increased muscle mass and a corresponding reduction in body fat (Van Laere et al., 2003).

Collectively, these studies highlight the important role played by epigenetically regulated loci in contributing to heritable phenotypic variation, making them attractive targets for candidate gene association studies and also inclusion in genome-wide scans that incorporate imprinting/parent-of-origin effects in domestic livestock species.

## Imprinted Genes as Candidates for Genotype–Phenotype Association Studies in Domestic Livestock

Since the 1950s, intense selection for economically important production traits (such as feed efficiency, milk production, meat quality, and fertility) has resulted in remarkable rates of genetic improvement and has led to the development of several elite high-performance livestock populations, most notably the Holstein-Friesian dairy breed. Initially, systematic science-based improvement of domestic livestock used quantitative genetic evaluation of phenotypic data generated from managed populations or pedigrees, such that individual animals displaying increased performance (as estimated through breeding values) for desired traits were selected as candidate parents for subsequent generations. In the last two decades, however, there has been a paradigm shift in animal genetic improvement research involving data generated from molecular genetic markers, which has been concomitant with advances in genome sequencing and genotyping technologies, bioinformatics and biostatistics. SNPs and simple tandem repeat (STR) loci represent two of the most abundant DNA sequence polymorphisms within the mammalian genome and are the predominant genetic markers used in genotype-phenotype association studies. The methods that form the basis of these programs involve testing for associations between measured traits (qualitative or quantitative) and genetic marker genotypes. The genetic markers used can be distributed across the whole genome [i.e., genome-wide association (GWA) studies] or be situated within or proximal to genes selected for analysis *a priori* based on their biological function (i.e., candidate gene association studies; Ron and Weller, 2007; Bush and Moore, 2012). Animals carrying a marker allele(s) or genotype(s) known to associate with a desired complex phenotype (often referred to as ‘quantitative trait loci’) may be selected as parental candidates for subsequent generations; this approach underpinned marker-assisted selection (MAS) strategies that were proposed for the genetic improvement of domestic livestock populations (Weller and Ron, 2011).

There have been a number of genotype–phenotype association studies in domestic livestock that either incorporate imprinting effects in the statistical models used, or which have focused specifically on DNA sequence variation in known imprinted or candidate imprinted genes based on their imprinting status in other species (de Koning et al., 2000; Rattink et al., 2000; Magee et al., 2010a). Early studies based on STR genotypes uncovered parent-of-origin QTL for a series of phenotypic traits in pigs, sheep and cattle. For example, parent-of-origin QTL influencing body composition, carcass and meat quality traits, growth traits and reproductive traits in the F_2_ progeny of experimental cross-bred pig populations (Nezer et al., 1999; de Koning et al., 2000; Rattink et al., 2000; Holl et al., 2004). Interestingly, a theoretical approach to identifying parent-of-origin effects on body composition data (eye muscle area, rib fat, rump fat, and intramuscular fat percent) collected from ultrasonic measurements revealed that a mean of 28% of the total genetic variance for these traits was due to parent-of-origin effects (Tier and Meyer, 2012).

A recent comprehensive genome-wide scan in cattle that specifically included a parent-of-origin inheritance model identified 24 parent-of-origin QTL (six were significant at the 5% genome-wide level and 18 were significant at the 5% chromosome-wide level) distributed across 15 bovine autosomes influencing growth and carcass traits; two of these QTL encompassed the bovine imprinted *GNAS* and *PEG3* genes (Imumorin et al., 2011). Subsequent studies have revealed associations between SNPs in the bovine *PEG3* and *GNAS* genes and growth-related traits, calving and fertility traits and animal health traits (e.g., somatic cell count, a marker of mastitis infection and susceptibility). Collectively, these results suggest that the *GNAS* and *PEG3* loci play an important role in bovine growth and development, fertility and health (Magee et al., 2010b; Sikora et al., 2011).

Additional studies revealing associations between imprinted loci and livestock production traits include the imprinted bovine *IGF2* and *IGF2R* genes and meat quality, milk production and growth traits in beef and dairy cattle populations (Flisikowski et al., 2007; Goodall and Schmutz, 2007; Bagnicka et al., 2010; Sherman et al., 2010; Berkowicz et al., 2011, 2012), although some authors contend that the *IGF2* associations with milk yields may be due to SNP alleles that are in linkage disequilibrium (LD) with neighboring variants in the proximal *INS* (insulin) gene, which contributes to the regulation of lactation (Akers, 2006; Berkowicz et al., 2011).

Associations between SNPs at the mammalian *DLK1-DIO3* imprinted gene cluster and production traits such as growth, fatness and body composition have also been reported in pigs (Kim et al., 2004; Oczkowicz et al., 2011) and cattle (Magee et al., 2011). These findings support the important role of this imprinted cluster in regulating mammalian growth. Notably, a recent survey of SNPs in the imprinted paternally expressed/maternally imprinted *DIO3* gene—which is involved in thyroid metabolism and has been shown to be highly expressed in uterine tissues in humans and rodents—was associated with fertility traits in pigs. It has been proposed that *DIO3* influences porcine fertility through the regulation of placental and/or fetal growth (Coster et al., 2012).

Examples of imprinted SNP-phenotype associations in pigs, sheep and cattle are listed in **Table 1**.

**Table 1:** Examples of associations between DNA sequence polymorphisms in known livestock imprinted genes and phenotypic traits.

Gene symbol/ Alias	Gene name	Encoded gene product function	Expressed allele	Species in which gene is imprinted	Phenotypic trait associations	Reference(s) for trait associations
*DIO3*	Deiodinase, iodothyronine, type III	Thyroid hormone regulation	Paternal	Pigs	Fertility traits	Coster et al. (2012)
*DLK1*	Delta-like homolog	Developmental growth factor; putative role in neuroendocrine differentiation; the purported effector protein in the development of the callipyge phenotype	Paternal	Pigs, sheep	Muscle hypertrophy; fat deposition; feed efficiency	Freking et al. (2002), Smit et al. (2003), Kim et al. (2004)
*DLX5*	Distal-less homeobox 5	A transcription factor involved in osteoblast differentiation and bone development	Maternal	Pigs	Carcass traits	Cheng et al. (2008)
*GNAS*	Guanine nucleotide-binding protein subunit alpha	Guanine nucleotide-binding proteins (G proteins) act as regulators in various signal transduction systems; forms part of the *GNAS* imprinting domain	Maternal; tissue-specific and developmental stage-specific paternal expression reported	Cattle, sheep	Growth traits; fertility traits; milk traits	Sikora et al. (2011), Oczkowicz et al. (2013)
*GRB10*	Growth factor receptor-bound protein 10	Signal transduction; interacts with insulin receptors and insulin-like growth factor receptors	Maternal	Cattle; sheep	Milk traits; body conformation traits	Magee et al. (2010b)
*IGF2*	Insulin-like growth factor 2	Positive regulator of cell division and mammalian growth and development	Paternal	Cattle, pigs, sheep	Growth traits; meat quality; millk production	Van Laere et al. (2003), Goodall and Schmutz (2007), Berkowicz et al. (2011)
*IGF2R*	Insulin-like growth factor 2 receptor	Non-mitogenic receptor for the IGF-II protein and transport of mannose-6-phosphate tagged proteins to lysosome	Maternal	Cattle, pigs, sheep	Growth traits	Berkowicz et al. (2012)
*MAGEL2*	MAGE-like 2	Putative regulator of neuronal development	Paternal	Cattle, pigs	Carcass traits; fertility traits	Guo et al. (2012), Jiang et al. (2014)
*MEG3/GTL2*	Maternally expressed gene 3/Gene trap locus 2	A non-coding RNA transcript that has been implicated in the regulation of *DLK1* expression, possibly through RNAi mechanisms.	Maternal	Cattle, pigs, sheep	Muscle hypertrophy; fat deposition; feed efficiency; growth traits; body conformation traits	Freking et al. (2002), Smit et al. (2003), Magee et al. (2011)
*MEG8*	Maternally expressed gene 8	A non-coding RNA transcript. Function not fully determined; implicated in the ovine callipyge phenotype.	Maternal	Cattle, sheep	Muscle hypertrophy; Fat deposition; Feed efficiency; Growth traits; Body conformation traits	Freking et al. (2002), Smit et al. (2003), Magee et al. (2011)
*NESP55*	Neuroendocrine secretory protein 55	Encodes a neuroendocrine secretory protein of largely unknown function; forms part of the *GNAS* imprinting domain	Maternal	Cattle, pigs	Growth traits; fertility traits; milk traits	Sikora et al. (2011)
*PEG3*	Paternally expressed gene 3	A role in cell proliferation and p53-mediated apoptosis	Paternal	Cattle, pigs, sheep	Fertility traits	Magee et al. (2010b)

*Examples experimentally validated imprinted genes in cattle, pigs, and sheep and their reported associations with complex production traits.*

## The Effects of Imprinted Gene Expression on Phenotype

**FIGURE 3:**
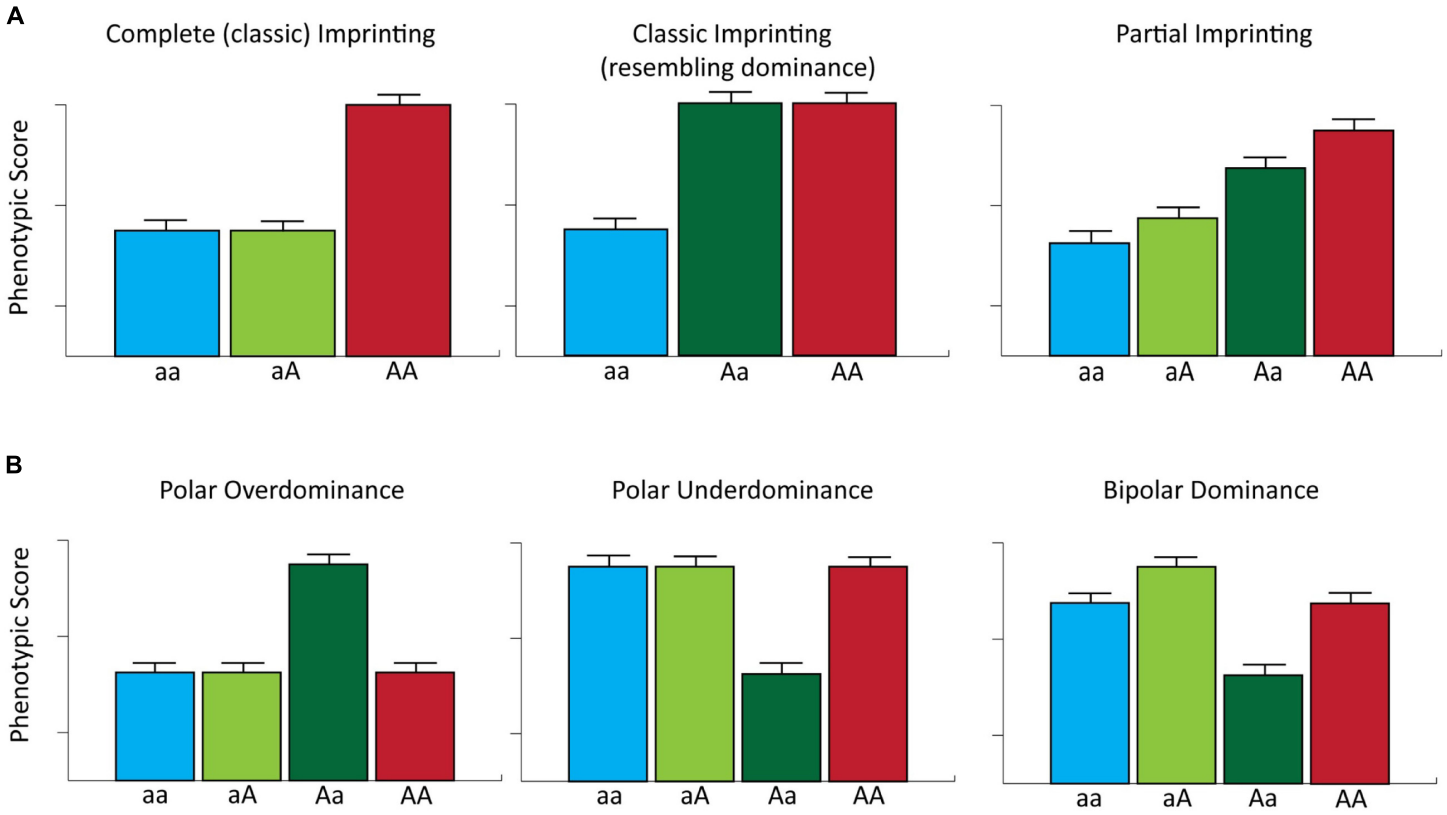
**Genomic imprinting and parent-of-origin effects on complex phenotypes. (A)** The phenotypic effects of complete and partial imprinting are considered for a single locus with two alleles. For complete imprinting, the first listed allele represents the expressed allele and the *A* allele has a greater effect on phenotype relative to the *a* allele. Note that in this example the *Aa* heterozygote displays a phenotypic score that resembles that expected for a locus with a dominance effect. For partial imprinting, *aA* and *Aa* represent reciprocal heterozygote genotypes, where the first listed allele is fully expressed and the second listed allele is partially expressed. In addition, the ‘*A*’ allele has the greatest effect on phenotype. Partial imprinting results in the generation of four potential phenotypic classes. **(B)** The phenotypic effects of a single locus for which there are two alleles displaying polar overdominance, polar underdominance and bipolar dominance modes of inheritance [modified from Lawson et al. (2011)].

The documented biological roles of imprinted genes in regulating mammalian growth and development together with the accumulating genotype–phenotype association data in domestic livestock species/populations, suggests that loci subject to genomic imprinting represent an important reservoir of genetic variation that may be exploited in selective breeding programs (Ruvinsky, 1999). However, genomic imprinting raises several interesting theoretical considerations for genotype–phenotype association studies. For example, classic imprinted gene expression (i.e., complete parent-of-origin monoallelic expression) is expected to generate patterns of phenotypic expression whereby phenotype is solely determined by the expressed allele. Consequently, classically defined imprinted loci with two alleles can be regarded as being functionally hemizygous (Bartolomei and Tilghman, 1997). This reduces the number of phenotypic classes at such loci from three (as expected under an additive genetic model) to two such that the heterozygote class is functionally equivalent to one of the two homozygote classes. It is important to note that for loci exhibiting complete imprinting, heterozygous individuals expressing the allele with the greatest phenotypic effect may display similar phenotypic scores to those traits controlled by loci with dominance effects (**Figure 3**). However, for many imprinted loci, transcriptional silencing is only partial (Khatib, 2007), which can generate functional differences between reciprocal heterozygotes (i.e., heterozygous individuals that have inherited the same allele from different parents) and can lead to four potential phenotypic classes (Spencer, 2000, 2009; **Figure 3**).

Furthermore, different forms of parent-of-origin effects can also generate phenotypic differences between genotype classes at the same locus. For example, polar overdominance, as exemplified by the callipyge phenotype, can result in phenotypic differences between reciprocal heterozygotes; in addition, under a model of polar overdominance, one of the heterozygous states will display a phenotypic value greater than all three other genotypes, which themselves show no differences in phenotypic values. Conversely, a model of polar underdominance, whereby one of the two reciprocal heterozygotes has a phenotypic value less than all three other phenotypically equivalent genotypes, has been reported in mice (Wolf et al., 2008). Finally, bipolar dominance can exist at imprinted loci such that one heterozygote displays larger phenotypic values and the other heterozygote exhibits lower phenotypic values than both homozygotes, which have the same phenotypic value (Wolf et al., 2008; **Figure 3**).

## Genomic Imprinting in the Era of Genome Selection

For many industrialized countries, the original MAS concept for livestock breeding has been supplanted by ‘genome-wide selection’ or ‘genomic selection’ using 1000s of genetic markers distributed across the genome. Genomic selection in livestock was originally proposed by Meuwissen et al. (2001) more than a decade ago; but has only been applied practically since the advent of high-density, pan-genomic livestock SNP genotyping arrays within the last 8 years. Genomic selection uses a genome-wide panel of dense markers so that all QTL are likely to be in LD (i.e., the non-random association of alleles at different loci) with at least one of the assayed SNPs. The genomic selection process involves the generation of genome-wide genotypic data for a large reference population of animals for which accurate phenotypic data are available. The resulting data serves as a reference for the development of statistical models that estimate the effect of each SNP with the trait(s)-of-interest, leading to the formulation of a predictive equation to estimate a genomic breeding value (GBV; i.e., the additive genetic component that is transmitted to the next generation). The predictive equation can then be used to impute the GBVs of additional animals as required (Goddard and Hayes, 2009; Goddard et al., 2010). This approach has been extremely successful, particularly for genetic improvement of dairy cattle and is rapidly becoming the method of choice for commercial breeding of beef cattle, pigs, and other livestock populations (Varona et al., 2015).

Genomic selection strategies are largely unconcerned with knowledge of the genes and causal variants that directly affect phenotypes (Snelling et al., 2013). However, several authors have recently argued for the refinement of genomic selection methods by incorporating all relevant genetic information that predict future phenotypes (i.e., the performance over its lifetime) rather than GBVs, including epigenetic patterns of gene expression (Gonzalez-Recio, 2011; Hayes et al., 2013). Such refinement might consider weighting imprinted gene-associated SNPs in genomic selection models according to expected effects on gene products during early development and across the whole lifespan of an individual (Snelling et al., 2013).

## Conclusion

The phenomena outlined above, demonstrate that imprinting parent-of-origin effects may complicate traditional quantitative genetic models used in phenotypic association studies. This review illustrates that imprinted gene expression can have a major effect on phenotypic traits in domestic livestock populations. Furthermore, imprinting is an important factor to consider in the models used for future the genetic improvement of domestic livestock for those genomic regions where imprinted gene expression is known to occur and to affect economically important traits included in the selection index.

## Conflict of Interest Statement

The authors declare that the research was conducted in the absence of any commercial or financial relationships that could be construed as a potential conflict of interest.

## Acknowledgments

We gratefully acknowledge the funding support from the University of Connecticut, the Irish Department of Agriculture, Food and the Marine Research Stimulus Program (project numbers RSF-06-406, RSF-06-0353, and RSF-06-0409**)** and from Science Foundation Ireland Investigator Grants awarded to DEM (Nos: SFI/01/F.1/B028 and SFI/08/IN.1/B2038) and CS (Nos: 02/IN.1/B49 and 08/IN.1/B1931).
